# Supplementary material for: Identification of photoperiod-induced specific miRNAs in the adrenal glands of Sunite sheep (Ovis aries)
Source: Front Vet Sci. 2022 Jul 22;9:888207. doi: 10.3389/fvets.2022.888207 (PMC9354845; doi:10.3389/fvets.2022.888207)
Supplement: Supplementary file 1 [file Data_Sheet_1.ZIP › Supplementary file/miRDeep2 file.zip!/miRDeep2 file/Novel_Structure/SPLP42A3/Novel_60.pdf]

Provisional ID	:	NC_019461.2_12223
Score total	:	1.9
Score for star read(s)	:	-1.3
Score for read counts	:	0
Score for mfe	:	1.6
Score for randfold	:	1.6
Score for cons. seed	:	
Total read count	:	13
Mature read count	:	13
Loop read count	:	0
Star read count	:	0

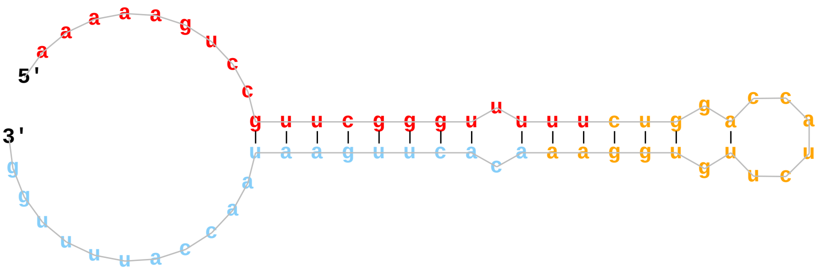

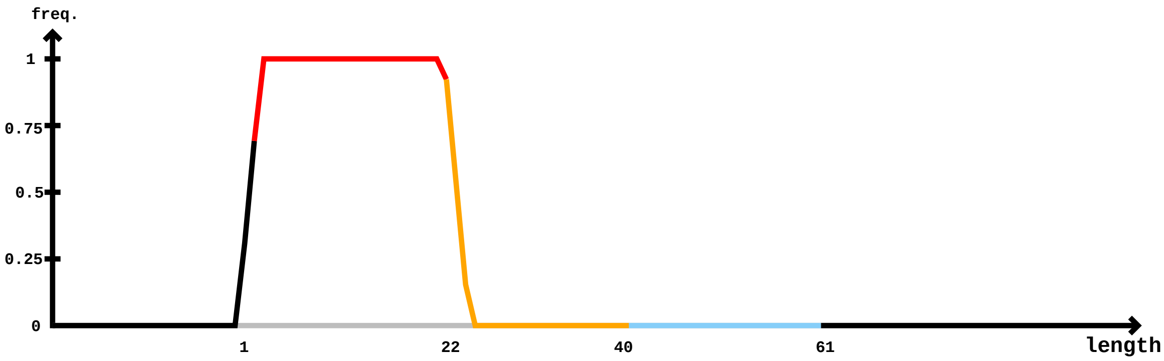

Mature Star

```
5'- gggauguauuagguuggccaaaaaguccguucggguuuuucuggaccaucuuguggaaacacuugaauaaccauuuuggccaaccccguacuuuguaguacauacacacgu   -3'    exp
    .(..(((((..((((((((((((.(...((((((((.((((((.(......).)))))).))))))))...).))))))))))))..(((((....))))))))))..)..     reads      mm         sample
    .................caaaaaguUcguucggguuuu.........................................................................     1          1          seq
    .................caaaaaguUcguucggguuuuu........................................................................     3          1          seq
    ..................aaaaaguUcguucggguuuuu........................................................................     5          1          seq
    ...................aaaaguccguucggguuuuu........................................................................     1          0          seq
    ...................aaaaguccguucggguuuuuc.......................................................................     1          0          seq
    ...................aaaaguUcguucggguuuuucu......................................................................     2          1          seq
```
